# Parent-of-origin effects orchestrate transcriptional reprogramming and epigenetic regulation of seedling vigor heterosis in triploid loquat

**DOI:** 10.3389/fpls.2025.1698577

**Published:** 2025-10-20

**Authors:** Chi Zhang, Ting Yuan, Shiqi Guo, Jiangbo Dang, Guolu Liang, Xiuhong Gou, Qigao Guo, Di Wu

**Affiliations:** ^1^ Key Laboratory of Agricultural Biosafety and Green Production of Upper Yangtze River (Ministry of Education), College of Horticulture and Landscape Architecture, Southwest University, Chongqing, China; ^2^ State Cultivation Base of Crop Stress Biology for Southern Mountainous Land of Southwest University, Academy of Agricultural Sciences of Southwest University, Chongqing, China

**Keywords:** loquat, triploid heterosis, parent-of-origin effects, allele-specific expression, allele-specific methylation, circadian clock, woody perennial

## Abstract

Triploid breeding is a promising avenue for generating seedless varieties with enhanced vigor, yet the underlying molecular mechanisms, particularly the relative contributions of hybridity, ploidy level, and parent-of-origin effects (POE), remain largely elusive in perennial fruit crops. This study focuses on loquat (*Eriobotrya japonica*), a highly heterozygous woody perennial, to explore the molecular mechanism of triploid seedling vigor heterosis. RNA-seq across a series of reciprocal diploid (2x), triploid (3x) and tetraploid (4x) hybrids with clear genetic background revealed POE as the predominant driver of triploid heterosis at the transcriptomic level. Specifically, 784 POE-responsive differentially expressed genes (DEGs) were identified between paternal-excess [3x(p)] and maternal-excess [3x(m)] triploids, exceeding the effects of ploidy (218–652 DEGs) and hybridity (8–90 DEGs). For an in-depth investigation, reciprocal crosses between L2 and L4 were further selected for an integrated transcriptome, allele-specific expression (ASE), and allele-specific methylation (ASM) analysis. Our findings demonstrate that POE orchestrates multilayered regulation, including (i) coordinated upregulation of vigor-related pathways (i.e., photosynthesis, starch metabolism, plant circadian rhythm) in 3x(p); (ii) a dual ASE pattern with maternal bias in gene quantity but paternal enhancement in levels, including five paternally expressed imprinted genes (PEGs); (iii) non-classical epigenetic regulation where paternal gene body hypermethylation (mCG) paradoxically enhances transcription, especially in circadian clock genes. Finally, qRT-PCR-based diurnal expression across all crosses validated that POE-dependent reprogramming of key circadian oscillator genes (*EjCCA1*, *EjLHY*, *EjGI*, *EjTOC1*), suggesting optimized metabolic efficiency through circadian clock modulation might contribute to enhanced vigor in 3x(p) hybrids. This study provides fundamental insights into the dosage-sensitive gene networks and epigenetic regulation underlying POE-driven heterosis in woody perennials, advancing polyploid heterosis theory and offering novel targets for genetic improvement.

## 1 Introduction

Polyploidy, referring to the state where an organism has more than two sets of chromosomes, is a widespread evolutionary force in plants kingdom and is also a powerful tool in crop breeding (Li and Luo, 2024). Triploid plants, with three chromosome sets, typically exhibit polyploid heterosis (enhanced vigor) and infertility. These characteristics make them highly suitable for breeding seedless varieties of fruit crops such as loquat (*Eriobotrya japonica*). This fruit crop has a cultivation history for over 2100 years and is now widely grown in more than 30 countries for its freshness and medicinal values (Lin et al., 1998; Su et al., 2021). Beyond seedlessness, triploid loquat typically exhibits enhanced vigor, including higher plant height, greater biomass accumulation, stronger stress resistance and elevated photosynthetic efficiency. These early growth advantages are particularly critical for loquat because the development of seedlings has significant impact on long-term performance and overall productivity (Liu et al., 2019, 2021; Xu et al., 2023). However, the underlying molecular mechanisms, particularly the relative contributions of hybridity, ploidy level, and parent-of-origin effects (POE), remain largely unknown in loquat.

Although the relative contributions of these three factors to triploid heterosis remain debated and context-dependent, recent studies have provided important insights into their individual roles in other plant systems. Studies in *Arabidopsis thaliana* (Miller et al., 2012) and maize (Yao et al., 2013) indicate that the ploidy level itself has minimal effects on triploid heterosis, while parental genome dosage and hybridity serve are the primary driving factors. To disentangle these factors, Fort et al. (2016) generated isogenic reciprocal triploids in *Arabidopsis*, revealing reciprocal differences in heterosis for rosette size, with positive effects in paternal excess triploids [3x (p), from diploid (2x) × tetraploid (4x)] and negative effects in maternal excess triploids [3x (m), from 4x × 2x]. This indicates that parental dosage alone can induce heterosis and highlights the role of parent-of-origin effects (POE) in regulating phenotypic outcomes. However, these contributions vary depending on trait. For example, in sugar beet (*Beta vulgaris*), hybridity mainly drives yield-related traits, whereas paternal genome dosage effects are more pronounced in vegetative traits such as leaf area and plant height (Hallahan et al., 2018). These POEs are not confined to annual species and have significant practical implications in perennial horticultural breeding. In apple (*Malus domestica*), the direction of the cross has been shown to directly impact the production rate of triploids (Sedov et al., 2014). Similarly, in citrus, parental choice is a key determinant for agronomic traits, ranging from the duration of the juvenile phase (Aleza et al., 2012) to the ultimate fruit quality parameters including acidity and sugar content (Ahmed et al., 2020). These insights collectively emphasize the trait- and species-specific nature of triploid heterosis, underscoring the necessity to explore underlying molecular mechanisms in varied plant systems.

At the molecular level, the unbalanced 2:1 parental genome ratio in triploid hybrids creates a unique regulatory paradigm, in which allele-specific expression (ASE) becomes common. This genomic dosage imbalance often leads to one parental allele being preferentially expressed over the other, resulting in allelic asymmetry that serves as the key molecular basis for POE (Fort et al., 2017; Castillo-Bravo et al., 2022; Li and Luo, 2024). The phenotypic consequences of this asymmetric expression are exemplified in triploid *Arabidopsis*, where paternal genome excess can alter expression of genes in starch metabolic pathways, leading to increased starch content and enhanced growth vigor (Miller et al., 2012). Similarly, core circadian clock genes exhibit different expression patterns in triploid hybrids, and these altered rhythms are directly related to growth heterosis (Ni et al., 2009; Miller et al., 2012), while genes related to nutrient reservoir activity show POE-dependent expression changes that contribute to seed size differences between reciprocal triploids (Fort et al., 2017). These POE-dependent expression patterns are frequently governed by epigenetic regulation, where DNA methylation can silence or activate specific parental alleles (Donoghue et al., 2014). Genomic imprinting, which is a form of this regulation caused by parent-specific methylation leading to monoallelic expression, represents an extreme form of this regulation and has become a key mechanism of POE in triploids (Donoghue et al., 2014).

Recent studies have employed high-throughput transcriptome and bisulfite sequencing to unravel intricate POE-dependent expression patterns and their epigenetic regulation. These efforts have primarily focused on the roles of ASE and DNA methylation in triploid heterosis across model plants and annual crops with simplified genetic backgrounds (Fort et al., 2017; Castillo-Bravo et al., 2022; Li et al., 2024). However, for perennial fruit trees with highly heterozygous backgrounds, studies on how hybridity, ploidy level, and POE interplay through gene expression and epigenetics remain scarce. Addressing this gap is essential for unraveling the genetic and epigenetic foundations of triploid heterosis, enabling optimized parent selection, molecular marker development, and breeding of elite seedless varieties with enhanced performance.

Building upon our established observation of significant seedling vigor heterosis in 3x(p) hybrids, particularly in plant height (Zhang et al., submitted for publication), this study utilized a series of reciprocal 2x, 3x, and 4x hybrid groups with clear genetic background to dissect the molecular mechanism underlying triploid heterosis. To this end, we first employed RNA-seq analysis across all groups to quantify the relative contributions of hybridity, ploidy level, and POE to triploid heterosis at the transcriptomic level. Subsequently, reciprocal crosses between L2 and L4 were further subjected to in-depth investigation, integrating transcriptomic, genome-wide ASE and allele-specific methylation (ASM) analyses to elucidate the molecular mechanisms underlying POE and their role in triploid heterosis. Altogether, these findings are expected to provide valuable insights into the complex genetic and epigenetic basis of triploid heterosis in woody perennials, offering novel targets for the genetic improvement of superior seedless loquat varieties.

## 2 Materials and methods

### 2.1 Plant materials and growth conditions

The plant materials investigated in this study consisted of a series of loquat groups. These included four parental lines: L2 (2n=2x=34) and L4 (2n=4x=68) derived from a red-fleshed cultivar ‘Longquan No. 1’; R2 (2n=2x=34) and R4 (2n=4x=68) derived from white-fleshed cultivar ‘Ruantiaobaisha’. The hybrid groups include (i) reciprocal 2x hybrids (R2×L2 and L2×R2) (by convention the maternal parent is listed prior to the paternal parent in a cross); (ii) reciprocal 3x hybrids consisted of maternal-excess triploids [3x(m): R4×R2, R4×L2, L4×R2, and L4×L2] and paternal-excess triploids [3x(p): L2×L4, R2×R4, L2×R4, and R2×L4]; (iii) reciprocal 4x hybrids (R4×L4 and L4×R4). In addition to the F1 hybrids, progeny from the self-pollination of each parental line (L2, L4, R2, and R4) were also generated as parental controls. The detailed methodology for crosses, confirmation of ploidy levels and hybrid authenticity has been described in our previous work (Zhang et al., submitted for publication). All seedlings were cultivated in soil pots under controlled greenhouse conditions, maintaining a 16-hour light/8-hour dark photoperiod. After 6 months, seedlings were transplanted to the Polyploid Loquat Germplasm Nursery of Southwest University (Beibei District, Chongqing, China).

### 2.2 Transcriptome analysis

For transcriptome sequencing, young, fully expanded leaves were collected from all hybrid groups and their corresponding parental groups. Our sampling strategy was designed to capture a transcriptomic representative of each group and minimize the effects of individual variation. For each hybrid and parental line, a total of nine individual plants were selected. These nine individuals were then randomly assigned to three biological replicates, with each replicate consisting of an equal amount of pooled leaf tissue from three distinct plants. Total RNA was then extracted from these pooled samples using the EASYspin Plant RNA Extraction Kit (RN09, Aidlab, Beijing, China). Following extraction, rRNA was depleted using the Ribo-ZeroTM Magnetic Kit (Epicentre, Madison, WI, USA), and stranded cDNA libraries were sequenced on an Illumina HiSeq X-Ten platform (RRID: SCR_016385, Parson Biotechnology Co., Ltd. Shanghai, China). Raw read sequences were deposited in the NCBI Sequence Read Archive (accession number PRJNA1268036). After quality filtering, clean reads were mapped to the same *Eriobotrya japonica* reference genome used in the Whole-Genome resequencing analysis, utilizing HISAT2 (version 2.2.1, RRID: SCR_015530). Gene expression was quantified as Fragments Per Kilobase of transcript per Million mapped reads (FPKM) using Salmon (v0.8.2, RRID: SCR_017036). Differentially expressed genes (DEGs) were identified using the DESeq2 package (v1.30.1, RRID: SCR_015687), applying thresholds of an absolute log_2_-transformed fold change ≥ 1 and a P-value < 0.05. Functional annotations and pathway enrichment analyses of the identified DEGs were conducted using the Gene Ontology (GO, RRID: SCR_002811) (http://geneontology.org/) and Kyoto Encyclopedia of Genes and Genomes (KEGG, RRID: SCR_012773) databases(http://www.genome.jp/kegg/).

### 2.3 Whole-genome bisulfite sequencing and DNA methylation analysis

**Figure 1:**
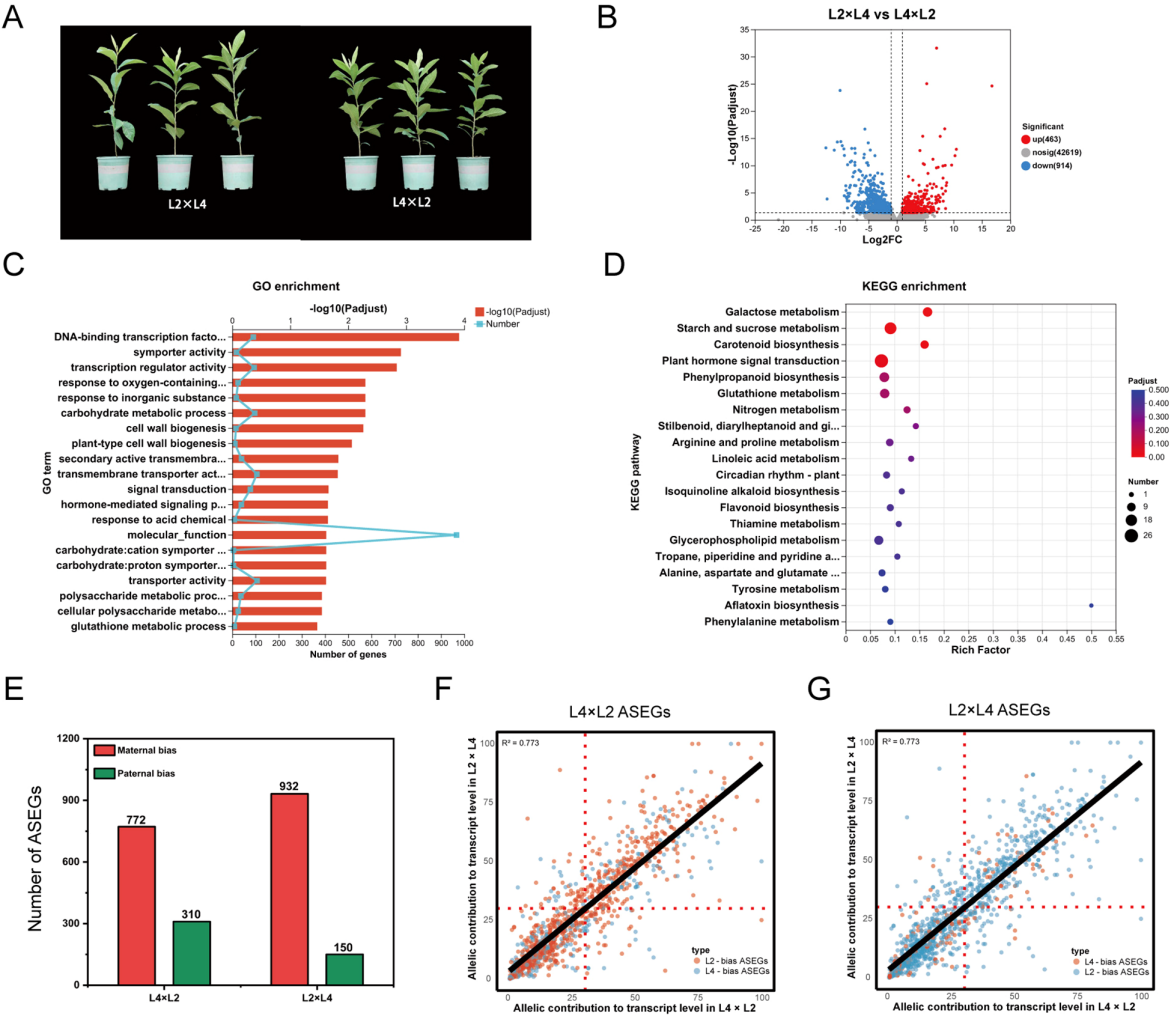
POE-driven phenotypic, transcriptomic, and allele-specific expression (ASE) divergence in L2×L4 [3x(p)] and L4×L2 [3x(m)] reciprocal crosses. **(A)** Phenotypic comparison of 2-year-old representative plants. B-D, Transcriptomic analysis including **(B)** volcano plot of DEGs between the reciprocal crosses, **(C)** GO and **(D)** KEGG pathway enrichment analysis of the identified DEGs. **(E)** Quantification of allele-specifically expressed genes (ASEGs) in reciprocal 3x hybrids. The bars represent the number of genes with significant maternal or paternal expression bias in L4×L2 and L2×L4. **(F, G)** Scatter plots comparing the allelic contribution (%) to total transcript levels for all ASEGs in **(F)** L4×L2 and **(G) **L2×L4, respectively; Each point represents a single ASEG; The x-axis shows the expression level of an allele when maternally inherited, and the y-axis shows its expression when paternally inherited.

For WGBS and subsequent DNA methylation analysis, the reciprocal triploid crosses L2×L4 [3x(p)] and L4×L2 [3x(m)] were selected as representative. This pair was chosen due to their minimal genetic differences among all diploid-tetraploid crosses that amplifies POE signals, as determined by genome-wide SNP analysis in our previous work (Zhang et al., submitted for publication). Furthermore, they exhibited a distinct phenotypic contrast, with L2×L4 showing greater plant height than L4×L2 (**Figure 1A**), consistent with stronger heterosis in 3x(p) versus 3x(m) hybrids. A total of nine individuals from each cross were randomly pooled into three biological replicates (3 plants per replicate). Genomic DNA was extracted from the selected samples using the CTAB method. The purified DNA fragments were then subjected to bisulfite treatment using the EZ DNA Methylation-Gold Kit (Zymo Research). The WGBS libraries were sequenced on an Illumina platform (RRID: SCR_016385, Parson Biotechnology Co., Ltd. Shanghai, China), and raw data were deposited in the SRA (accession number: PRJNA1267249). Raw reads were filtered using FastQC (v0.11.9, RRID: SCR_014583) and Trimmomatic (v0.39, RRID: SCR_011848) to remove adapters and low-quality bases. Clean reads were then aligned to the *Eriobotrya japonica* ‘Jiefangzhong’ reference genome using Bismark (v0.20.0, RRID: SCR_005604) with default parameters. The methylation level for each cytosine in the mCG, mCHG, and mCHH contexts was calculated as the ratio of methylated reads to total reads covering that site. Only cytosines covered by at least 5 reads were considered for further analysis. Overall methylation levels for gene bodies were calculated for all annotated genes.

### 2.4 Allele-specific expression analysis

RNA-seq reads from the reciprocal hybrids (L4×L2 vs. L2×L4) were mapped to the reference genome using HISAT2 (v1.4.1, RRID: SCR_015530). To identify reads originating from each parental allele, we utilized the high-confidence SNP set previously identified between the parental controls. The ASEReadCounter tool in GATK (v4.1.8, RRID: SCR_001876) was used to count reads supporting each parental allele at informative SNP loci. For a gene to be considered for ASE analysis, it was required to contain at least 1 informative SNP covered by a minimum of 10 total reads across all biological replicates. For 3x hybrids, the expected allelic ratio is 2:1 (4x vs. 2x). A binomial test was performed to assess whether the observed read counts for the 2 parental alleles at each SNP significantly deviated from this expected 2:1 ratio. P-values were adjusted for multiple testing using the Benjamini-Hochberg method to control the false discovery rate (FDR). A gene was classified as exhibiting significant ASE (an ASEG) if at least 1 SNP within it had an FDR < 0.05. A gene was defined as paternally or maternally biased if the allele from the paternal or maternal parent, respectively, showed significantly higher expression level than expected under a 2:1 dosage ratio.

### 2.5 Allele-specific methylation analysis

To dissect the epigenetic mechanisms underlying ASE, we conducted an ASM analysis. The Bismark-aligned WGBS reads from hybrids (L4×L2 and L2×L4) were partitioned into parental-specific sets using SNPsplit (v0.3.4), which segregates reads based on the sequence at known heterozygous SNP loci. This process generated allele-resolved methylomes for both the L2- and L4-derived alleles within each cross. The methylation level for each cytosine was calculated separately for each parental allele. To quantify ASM, we focused on genomic regions (downstream, gene body, upstream) of the identified ASEGs. For each region within a gene, we calculated the ASM difference as the absolute difference in mean methylation levels between the L2 and L4 alleles. To assess the POE on ASM, the distributions of these ASM difference values were compared between the reciprocal hybrids (L4×L2 vs. L2×L4) using a Wilcoxon rank-sum test. The Integrative Genomics Viewer (IGV, RRID: SCR_011793) was used to visualize individual loci, displaying allele-specific RNA-seq coverage and DNA methylation patterns.

### 2.6 Quantitative reverse transcription PCR validation

The qRT-PCR was performed on cDNA synthesized from RNA extracted from all hybrid groups and their parental controls. Reactions were conducted using a SYBR Green-based master mix on a Bio-Rad CFX 96 Real-Time PCR Detection System (RRID: SCR_018064, Bio-Rad, Inc., CA, USA). To determine the expression levels of the relative genes, they were normalized to the internal reference gene and calculated using the 2^−ΔΔCt^ method. The qRT-PCR was employed for two distinct purposes. First, for a general technical validation of the RNA-seq data, 10 DEGs showing a range of expression patterns were randomly selected. Second, for a specific biological validation of our core hypothesis, a detailed diurnal expression analysis of key circadian clock genes was performed, with samples collected at ZT0, ZT6, ZT12, and ZT18. All reactions were conducted with 3 biological and 3 technical replicates. The sequences of all primer pairs are listed in **Supplementary Table 1**.

## 3 Results

### 3.1 Global transcriptome profiling of POE-driven gene expression changes in triploid loquat

To investigate the transcriptional basis of triploid heterosis in loquat, we performed RNA-seq analysis across all hybrid groups and parental controls. A total of 48 cDNA libraries yielded over 325.54 GB of high-quality clean data (Q30 > 94.32%) (**Supplementary Table 2**). The overall technical reliability of our RNA-seq dataset was validated through qRT-PCR analysis of randomly selected 10 DEGs, which showed strong correlation with RNA-seq results (**Supplementary Figure 1**).

**Figure 2:**
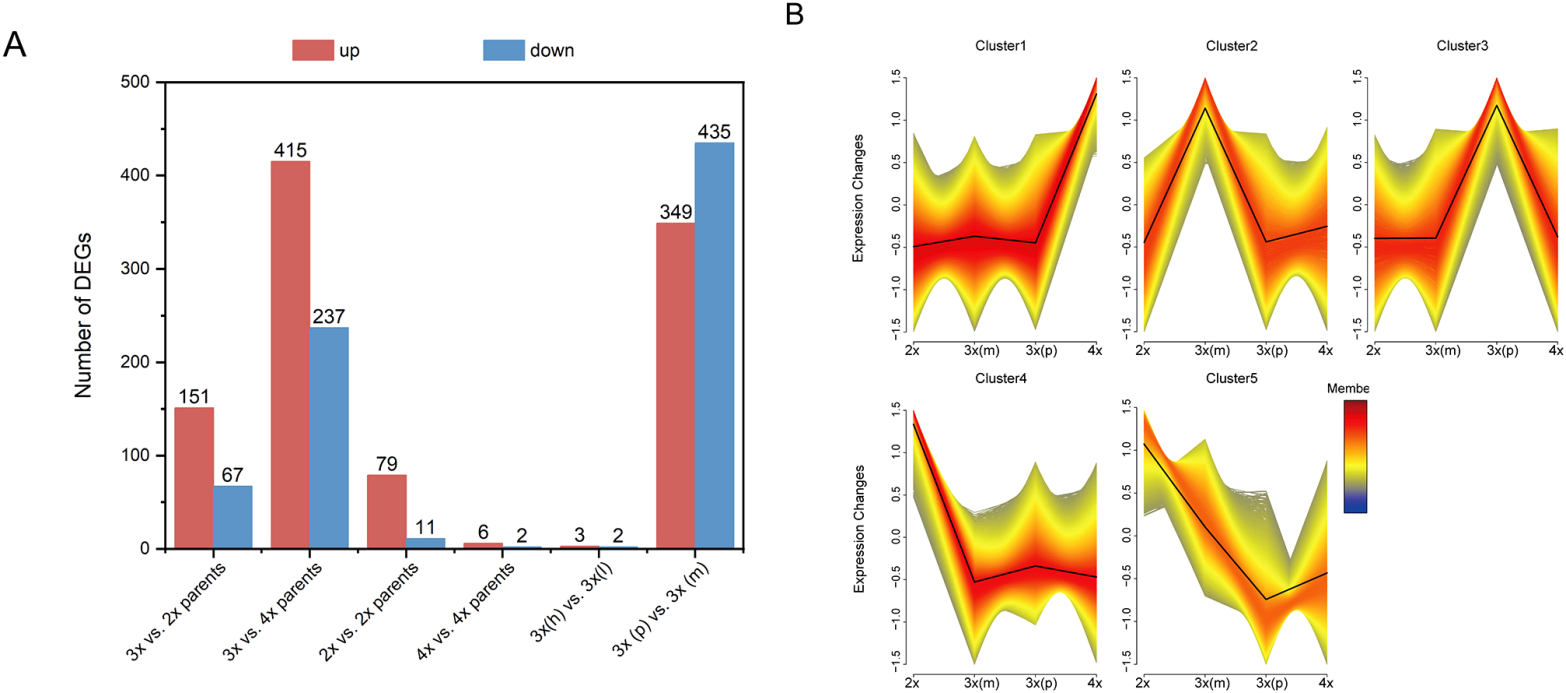
Global transcriptomic analysis of different loquat groups. **(A)** Number of upregulated (red) and downregulated (blue) differentially expressed genes (DEGs) in key combinations; The bar chart quantifies the DEGs number resulting from the ploidy effect (3x vs. 2x parents and 3x vs. 4x parents), the hybridity effect at 2x and 4x level (2x vs. 2x parents; 4x vs. 4x parents), the POE at 3x level [3x(p) vs. 3x(m)], the parental genetic distance effect [highly related 3x(h) vs. lowly related triploids 3x(l)]. **(B)** Five major expression profiles (cluster 1-5) of all DEGs identified by fuzzy c-means clustering across all groups; Each plot shows the standardized expression change (y-axis) across different groups (x-axis), with black line representing the mean expression trend for each cluster.

Systematic pairwise comparisons revealed substantial transcriptional reprogramming associated with parental genome dosage effects (**Figure 2A**). The most striking finding was that POE comparisons [3x(p) vs. 3x(m)] yielded that largest number of DEGs (784 genes), substantially exceeding the effects of ploidy level differences (3x vs. 2x parents: 218 DEGs; 3x vs. 4x parents: 652 DEGs) and the effect of hybridity within ploidy levels (2x vs. 2x parents: 90 DEGs; 4x vs. 4x parents: 8 DEGs). To assess the impact of parental genetic distance on transcriptional responses, we categorized 3x hybrids into 2 groups based on parental genetic distance, including 3x(h) derived from intra-cultivar crosses (i.e. L2×L4 and L4×L2) and 3x(l) derived from inter-cultivar crosses (i.e. L2×R4 and R4×L2). Notably, comparisons between triploids from genetically distant parents showed minimal transcriptional differences (5 DEGs), likely reflecting the dominant contribution of POE over genetic background effects in shaping global transcriptional profiles. Factorial contribution analysis revealed that POE dominated triploid heterosis at transcriptomic levels, as supported by the number of POE-responsive DEGs exceeding those of ploidy and hybridity effect alone.

To identify expression patterns associated with superior 3x (p) performance, all DEGs were subjected to fuzzy c-means clustering using Mfuzz (membership threshold = 0.7, fuzzification parameter = 2). This analysis revealed 5 distinct clusters, with Cluster 3 showing the most relevant profile where gene expression was highest in 3x(p) hybrids among all groups (**Figure 2B**). Consequently, the 4,688 genes in Cluster 3 were further partitioned into 10 sub-clusters (C1-C10) using K-means clustering for detailed functional analysis.

**Figure 3:**
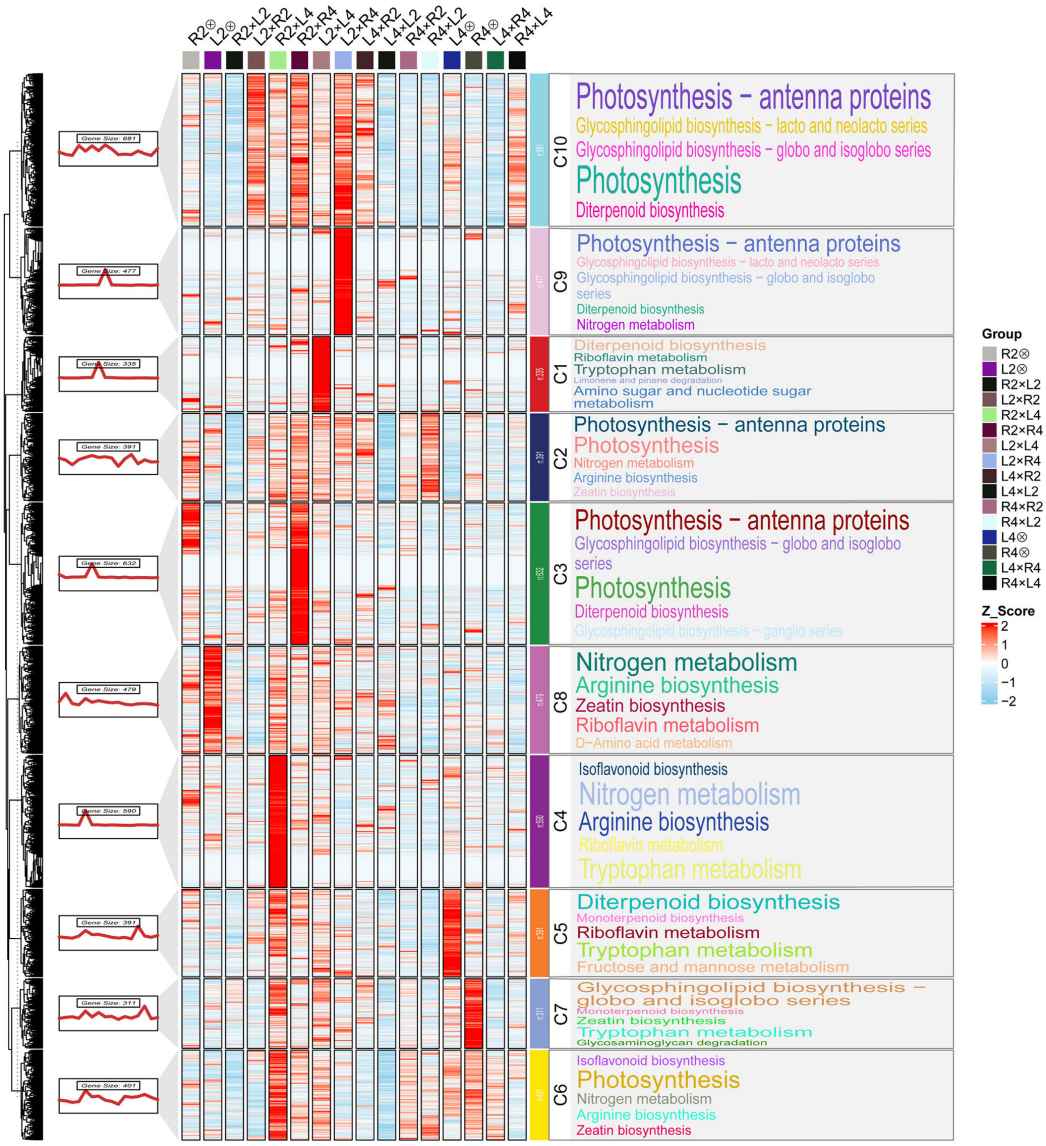
Heatmap and KEGG pathway enrichment of genes from Cluster 3. Cluster 3 genes, which exhibited the highest expression level in 3x(p), were further partitioned into 10 co-expression modules (C1–C10) by K-means clustering. The heatmap displays Z-score normalized expression of each gene across all samples for 10 co-expression modules; The color scale (Z-score) indicates relative expression level, with red representing high expression and blue representing low expression; The significantly enriched KEGG pathways within each module are visualized as word clouds. The font size of each term is proportional to the number of differentially expressed genes (DEGs) assigned to that pathway.

KEGG enrichment analysis of these sub-clusters revealed that multiple clusters (C1, C2, C3, C9, and C10) showed significant enrichment for photosynthesis-related pathways, including “Photosynthesis” (ko00195) and “Photosynthesis-antenna proteins” (ko00196) (**Figure 3**). This indicates that enhanced light energy utilization efficiency, especially through heightened activity of light-harvesting complexes, constitutes a potential determinant of heterosis in the 3x(p) (Friedland et al., 2019). Growth-promoting hormone pathways were also prominently enriched, with “Diterpenoid biosynthesis” (ko00904) for gibberellin synthesis significantly enriched in C10, C3, and C5, and “Zeatin biosynthesis” (ko00908) for cytokinin synthesis enriched in C2, C7, and C6. The coordinated action of these phytohormone pathways is essential for stem elongation and overall plant development, providing molecular support for observed triploid heterosis (Singh and Roychoudhury, 2022). Additionally, nitrogen metabolism pathways, particularly “Nitrogen metabolism” (ko00910) and “Arginine biosynthesis” (ko00220), were significantly enriched in C2, C8, C4, and C6, suggesting that 3x(p) possess improved nitrogen absorption, assimilation, and utilization efficiency, thereby providing ample precursors for protein and nucleic acid synthesis and facilitating plant growth (Zhao et al., 2018; Li et al., 2025). Furthermore, “Amino sugar and nucleotide sugar metabolism” (ko00520) was enriched in C1, directly supporting elevated photosynthetic efficiency by mediating the generation of nucleotide sugars, including ADP-glucose, which serves as a direct substrate for starch synthases (Feng et al., 2022). Collectively, the synergistic upregulation of DEGs within these metabolic and physiological pathways provides comprehensive molecular support for superior performance observed in the 3x(p) hybrids for heterotic seedling vigor traits.

### 3.2 Allele-specific expression analysis of POE mechanisms in L2×L4 and L4×L2 triploid hybrids

Building on the global transcriptome findings that identified POE as the primary driver of transcriptional variation, we focused on reciprocal triploids L2×L4 [3x(p)] and L4×L2 [3x(m)] for in-depth molecular analysis. This pair was selected as representative crosses because it had the smallest genetic differences among all diploid-tetraploid crosses, helping to minimize effects from other genetic background factors and better highlight POE. Phenotypic data further confirmed this, with L2×L4 showing greater plant height compared to L4×L2 plants (**Figure 1A**), consistent with the stronger heterosis in 3x(p) versus 3x(m) hybrids.

Comparative transcriptome analysis between L2×L4 and L4×L2 revealed substantial reprogramming, with 1,377 DEGs identified, including 463 upregulated and 914 downregulated (**Figure 1B**). This magnitude of transcriptional divergence between genetically similar reciprocal hybrids suggests that POE may drive the observed gene expression patterns (Donoghue et al., 2014). GO analysis showed significant enrichment in modules such as “DNA-binding transcription factor activity” (GO:0003700) and “transcription regulator activity” (GO:0140110), suggesting POE influences hybrid growth through hierarchical regulatory networks (Zhang et al., 2019). Further terms like “carbohydrate metabolic process” (GO:0005975) and “plant-type cell wall biogenesis” (GO:0009505) correlated with heterosis in plant height (**Figure 1C**). KEGG pathways were enriched in “starch and sucrose metabolism” (ko00500), “nitrogen metabolism” (ko00910), and “plant hormone signal transduction” (ko04075), indicating dosage effects on carbohydrate dynamics, nitrogen efficiency, and hormonal homeostasis (**Figure 1D**). These results align closely with the global transcriptome patterns in Section 3.1. Interestingly, the “plant circadian rhythm” pathway (ko04712) was also enriched, suggesting parental dosage modulates fundamental rhythms like growth timing and metabolic synchronization, potentially contributing to heterosis (Greenham and McClung, 2015). These transcriptome results validate the selection of this cross as a representative model for POE studies and implying dosage imbalances disrupt sensitive regulatory networks, as observed in *Arabidopsis* triploids (Fort et al., 2016). Overall, the asymmetric expression driven by POE prompted deeper investigation into underlying allelic mechanisms.

To uncover the allelic basis of POE-driven transcriptional differences, we conducted genome-wide ASE analysis. The analysis revealed a pronounced maternal dominance governing the number of allele-specifically expressed loci (ASEGs) (**Figure 1E**). In the L4×L2 cross, 772 ASEGs (9.44% of informative genes) exhibited a maternal bias to the L4 allele, whereas only 310 ASEGs (3.79%) showed a paternal bias to the L2 allele. This pattern was further reinforced in the reciprocal L2×L4 cross, with 932 ASEGs (11.40%) showing maternal biased to L2 versus 150 ASEGs (1.84%) with paternal bias to L4. These quantitative asymmetries demonstrate a predominant maternal role in allele selection, likely mediated by epigenetic marks that silence paternal homologs during early embryogenesis (Castillo-Bravo et al., 2022). However, delving deeper into allelic expression magnitude revealed a contrasting regulatory pattern (**Figures 1F, G**). Scatter plots showed L2-biased ASEGs (orange dots) clustered predominantly below the identity line, indicating higher expression of L2 allele when paternally inherited (L4×L2). Conversely, L4-biased ASEGs (blue dots) clustered primarily above the identity line, reflecting enhanced L4 allele expression when it is a paternal contributor (L2×L4). Maternal factors appear to dictate which allele is expressed, likely via an imprinting-like silencing mechanism, while the paternal contribution serves to amplify the expression of this chosen allele, possibly by reducing repression or activating its promoter. Ultimately, this intricate interplay represents not merely simple parental dominance, but a subtle molecular foundation for POE that underpins triploid heterosis by generating allelic asymmetries to optimize gene dosage in heterozygous contexts.

Genomic imprinting, a canonical POE mechanism characterized by strictly uniparental expression, represents an extreme form of this paternal enhancement (Reik and Walter, 2001). We applied stringent criteria (requiring consistent, significant uniparental bias in both reciprocal crosses, FDR < 0.05) and identified 6 candidates paternally expressed genes (PEGs; **Supplementary Table 3**). For example, Ej00021050 on chromosome 5 showed a strong paternal bias. In L4×L2, the paternally derived L2 allele accounted for approximately 95.5% of the transcripts. Conversely, in the reciprocal L2×L4, the paternal L4 allele dominated, contributing 62–69% of the transcripts. This POE-dependent expression, independent of allelic sequence, is a classic hallmark of paternal imprinting (Jiang et al., 2021). Functionally, the enrichment of these PEGs in metabolic regulators, such as starch-related enzymes (**Supplementary Table 3**), strongly implies their direct contribution to enhanced starch accumulation and growth in 3x(p) hybrids. However, the number of imprinted genes we identified in loquat leaf tissue is lower than the dozens reported in the seed tissues of model plants like *Arabidopsis* (Fort et al., 2017).Subsequent longitudinal studies are required to monitor these hybrid populations to maturity. Such research will be crucial to elucidating how parent-of-origin effects modulate pivotal agronomic traits, such as fruit yield and quality, and ultimately, to determine whether the molecular signatures of seedling vigor translate into enhanced lifelong crop performance. We propose this difference reflects the unique biological context of loquat as a perennial and highly heterozygous species. This finding ultimately underscores the necessity of developing species- and traits-specific POE models to advance fruit tree breeding.

### 3.3 Allele-specific DNA methylation associated with POE in L2×L4 and L4×L2 triploid hybrids

**Figure 4:**
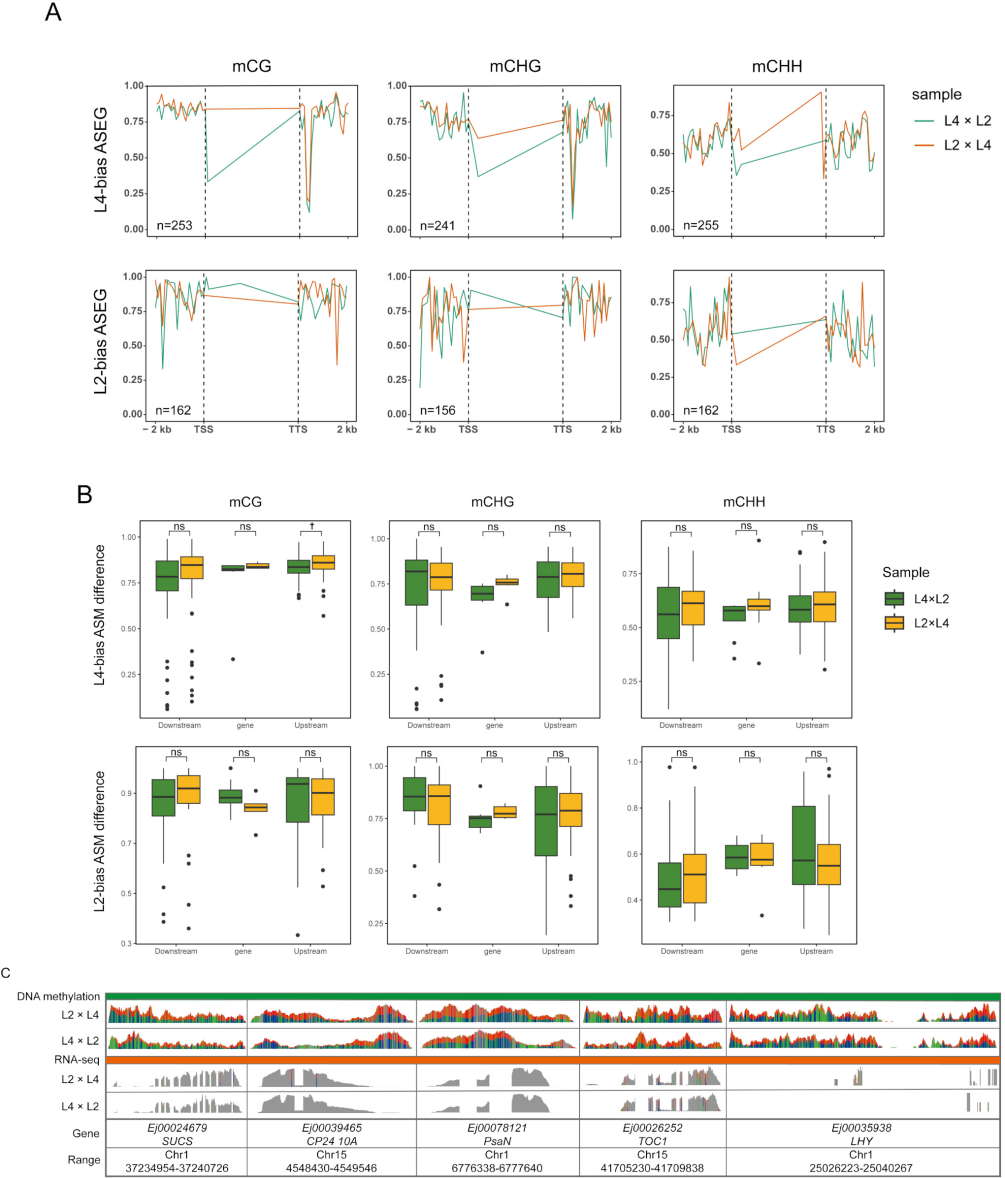
POE-driven allele-specific DNA methylation (ASM) and its effects on gene expression in L2×L4 and L4×L2 reciprocal crosses. **(A)** Metaplots of average DNA methylation profiles (mCG, mCHG, and mCHH) for the biased parental allele in L4-biased and L2-biased ASEGs; Profiles are shown across the gene body (TSS to TTS) and 2-kb flanking regions. **(B)** Boxplots comparing the ASM difference between L4×L2 (green) and L2×L4 (yellow) reciprocal hybrids; The comparison is shown for promoter, gene body, and downstream regions of L4-biased and L2-biased ASEGs; Boxes indicate the interquartile range and median; †*P* < 0.1; ns indicates not significant. **(C)** Genome browser view of allele-specific BS-seq (methylation) and RNA-seq (expression) signals at representative ASEG loci (*EjSUCS*, *EjCP24 10A*, *EjPsaN*, *EjTOC1*, *EjLHY*); The gray-shaded region indicates the gene body.

To investigate the epigenetic mechanisms underlying the POE- driven allelic expression patterns identified in our ASE analysis, we performed whole-genome bisulfite sequencing (WGBS) and conducted an integrated allele-specific methylation (ASM) analysis on the L4×L2 and L2×L4 reciprocal crosses. ASE analysis revealed context-specific parental dosage effects on gene body (TSS to TTS region) (**Figure 4A**). In the mCG context, paternal alleles consistently exhibited higher methylation than maternal alleles across both L4- and L2-biased ASEGs. This paternal dominance persisted in the mCHH context but reversed near the TTS in the 3’ region. In contrast, mCHG showed paternal dominance only in L4-biased ASEGs, indicating a more nuanced pattern. Therefore, a POE-specific methylation pattern was primarily observed in the mCG context, with high methylation of paternal alleles in the gene body region, a phenomenon known as gene body methylation (gbM) (Takuno and Gaut, 2012; Wang et al., 2015). Critically, this paternal-biased gbM showed a strong positive correlation with the enhanced expression levels of the paternal alleles.

Based on this, we performed ASM analysis to quantify methylation differences between L2 and L4 parental alleles within each cross (**Figure 4B**). In L4-biased ASEGs, the mCG methylation difference (ASM difference) in the promoter region of the paternal-excess hybrid (L2×L4) was significantly higher than that of its maternal-excess cross (L4×L2) (*P* < 0.1). This result indicates that in the L2×L4 cross, the promoter of the actively expressed L4 allele (from the paternal parent) is likely deeply hypomethylated, while the promoter of the suppressed L2 allele (from the maternal parent) is highly methylated, leading to a significant methylation difference. This pattern is consistent with classic epigenetic regulatory mechanisms, where the activation and silencing of allele expression are associated with promoter hypomethylation and hypermethylation, respectively (Donoghue et al., 2014).

In the gene body region, hypermethylation of paternal alleles showed a strong positive correlation with their enhanced expression levels. To validate this finding at a single-locus level, we visualized the ASE and methylation status of key functional genes involved in heterosis using IGV, including representative genes such as *EjPsaN* (photosynthesis), *EjCP2410A* (Photosynthesis- antenna proteins), *EjSUCS* (starch synthesis), and the core circadian clock genes *EjTOC1* and *EjLHY* (Ng et al., 2014; Lu et al., 2024). IGV snapshots consistently revealed that paternal alleles systematically exhibited higher transcript abundance than maternal alleles at the transcriptional level (**Figure 4C**). Parallel to this expression pattern, the gene body ASM profiles also showed a similar asymmetry. Specifically, higher-expressing paternal allele correlated with its hypermethylation, while lower-expressing maternal allele was hypomethylated. This observation was consistent with our ASE analysis (**Figure 4A**). Notably, the gene bodies of core circadian clock genes exhibited an asymmetry in mCG methylation. This modification was significantly present on the higher-expressing paternal alleles but was absent on the corresponding maternal alleles (**Supplementary Tables 3, 4**). In line with previous work (Lauss et al., 2018; Ng et al., 2014), these results indicate a possible facilitative role of gene body methylation in enhancing paternal allele expression in core circadian regulators, potentially driving heterosis through optimized diurnal rhythms.

### 3.4 Diurnal expression analysis of core circadian clock genes under POE Influence in triploid loquat

**Figure 5:**
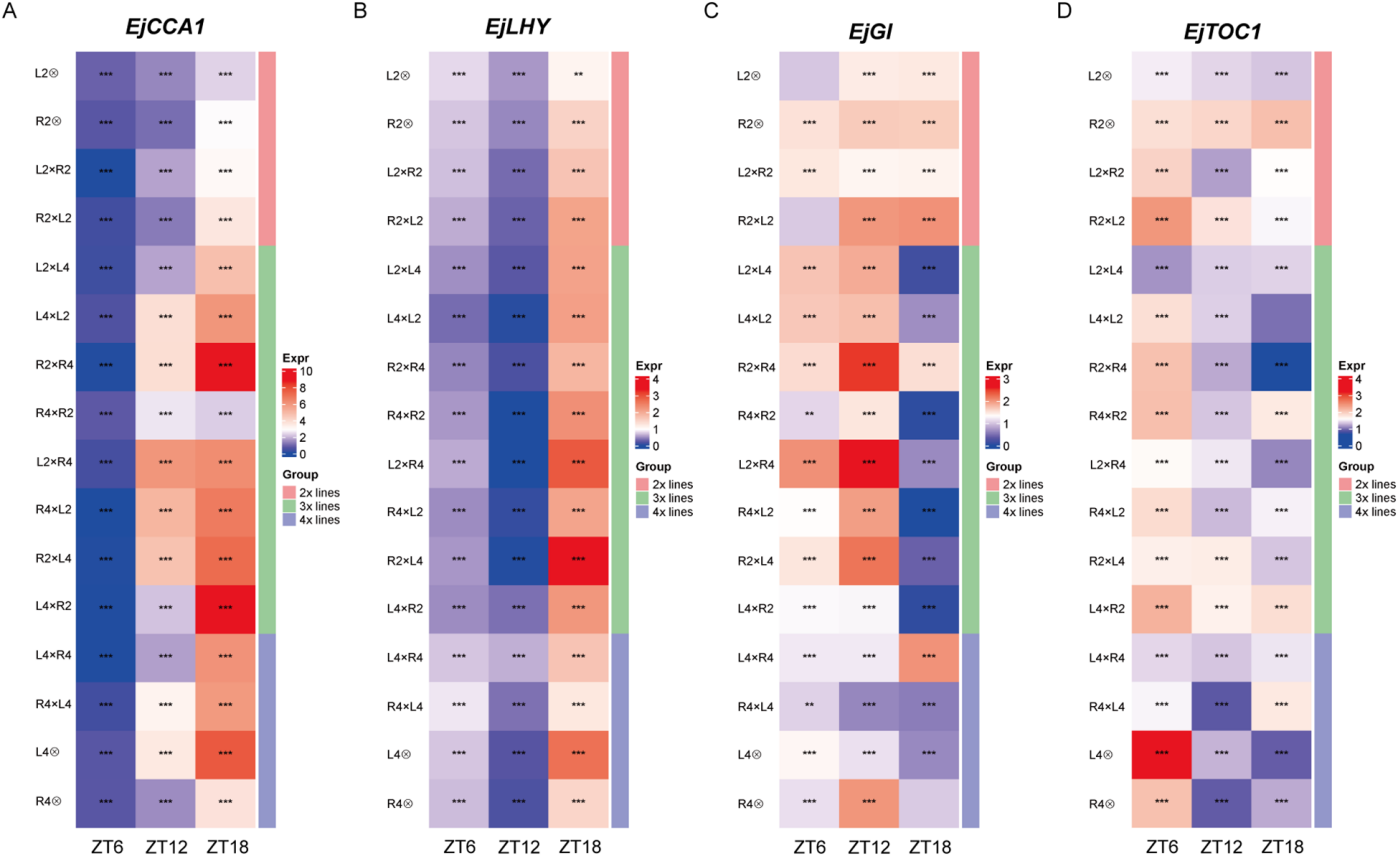
Diurnal expression profiles of core clock genes in loquat hybrid and parental groups. **(A–D)** Heatmaps showing the normalized transcript abundance for 4 core circadian clock genes, including **(A)** *EjCCA1*, **(B)** *EjLHY*, **(C)** *EjGI*, and **(D)** *EjTOC1*. Samples were collected at 4 Zeitgeber times (ZT0, ZT6, ZT12, ZT18). The color scale (Expr) indicates relative expression level, with red representing high expression and blue representing low expression. The vertical bar on the right (Group) categorized each line by ploidy level; Asterisks denote statistically significant differences (*P < 0.05, **P < 0.01, ***P < 0.001) in expression levels between the reciprocal triploid hybrid pairs (e.g., L2×L4 vs. L4×L2; R2×R4 vs. R4×R2) at each specific Zeitgeber time.

The epigenetic regulation of POE (Section 3.3), combined with the enrichment of the “plant circadian rhythm” pathway in POE-affected DEGs, positions the clock as a key downstream target of POE, leading us to hypothesize that parental dosage reprograms the core circadian clock. As a master regulator of plant physiology that coordinates fundamental processes like photosynthesis and starch turnover, we propose that its reprogramming mediates the observed heterosis in triploid seedling vigor, as supported by studies on clock-driven metabolic optimization and hybrid advantages (Izumi, 2019; Xu et al., 2022). To test this, we conducted the qRT-PCR-based diurnal expression of 4 central clock components, *EjCCA1*, *EjLHY*, *EjGI* and *EjTOC1* at Zeitgeber times (ZT) 0, 6, 12, and 18 (**Figure 5**).

In 2x and 4x groups, the four clock genes exhibited diurnal oscillations with a notable phase shift in loquat compared to canonical models like *Arabidopsis*, likely reflecting loquat’s subtropical adaptation (Greenham and McClung, 2015). Specifically, the putative morning-expressed gene *EjCCA1* and *EjLHY* showed inverted phases, with *EjCCA1* having its lowest expression (nadir) at ZT6 and transcript levels rising to a peak at the end of the light period or early evening, while *EjLHY* displayed a similar trend but with a delayed nadir to ZT12 (**Figures 5A, B**). Conversely, the putative evening-expressed genes *EjGI* and *EjTOC1* generally peaked earlier in the day (ZT6 or ZT12) before declining (**Figures 5C, D**). This conserved antiphase relationship, although inverted phase relative to model systems, confirms the functionality of the core feedback loop in loquat (Harmer et al., 2000; Filichkin et al., 2011; Hsu and Harmer, 2014).

However, this core oscillatory network was reprogrammed in triploid loquat, where imbalanced parental genome dosage manifested as significant POE-dependent alterations in expression level, phase and rhythmic pattern. These changes were most evident in comparisons between 3x(p) and 3x(m) hybrids. For *EjCCA1*, expression in 3x(p) (i.e., R2×R4) was dramatically elevated at ZT18 relative to its 3x(m) counterpart, exhibiting a strong, late-day upregulation specific to 3x(p) (**Figure 5A**). A similar, though less pronounced, paternal-excess-driven elevation was observed for *EjLHY* in R2×L4 vs. L4×R2 (**Figure 5B**). The remaining clock components also exhibited a strong POE. For *EjGI*, expression was maintained at a constitutively high level across all time points in the 3x(p) hybrid (L2×R4), while being severely and constitutively repressed in the 3x(m) hybrid (R4×L2) (**Figure 5C**). The reprogramming of *EjTOC1* expression was particularly obvious across all triploid reciprocal crosses, where paternal genome excess induced a general dampening of its rhythmic amplitude (about 40% reduction in L4×L2), which in turn produced the low and flattened profiles in 3x(p) like L2×L4, R2×R4, and L2×R4 (**Figure 5D**). In contrast, 3x(m) counterparts exhibited much more dynamic and often aberrant rhythms, such as a distinct peak at ZT6 in L4×L2 or at ZT12 in R4×R2. This opposing behavior between 3x(p) and 3x(m) triploids underscores a POE-dependent regulation, where the paternal genome dosage appears to exert a repressive or stabilizing effect on *EjTOC1* dynamics. Collectively, these findings demonstrate that POE in triploid loquats drive heterosis by reprogramming the central circadian clock, likely through optimizing metabolic efficiency, which establishes the clock as a key new target for perennials crop improvement (Ng et al., 2014).

## 4 Discussion

### 4.1 POE-driven transcriptional reprogramming underlies heterosis in triploid loquat

Our study demonstrates that POE is a primary driver of transcriptional reprogramming in triploid loquat, far exceeding the effect of ploidy or hybridity alone (**Figure 2A**). This finding aligns with studies in other plant systems, such as *Arabidopsis* and maize, where imbalanced parental contributions lead to non-additive gene expression patterns that are crucial for hybrid vigor (Fort et al., 2016; Yao et al., 2013; Wu et al., 2021; Li and Luo, 2024). Specifically, the 3x(p) exhibited a distinct transcriptional landscape characterized by the significant upregulation of DEGs enriched in vigor-related pathways, including photosynthesis, phytohormone biosynthesis (e.g. gibberellin and cytokinin), nitrogen metabolism, and starch synthesis (Cluster 3, **Figure 3**). These pathways are fundamental processes that support plant growth and biomass accumulation. The coordinated upregulation of these enriched pathways indicates the presence of a highly efficient metabolic network in 3x(p) hybrids. For instance, the increased expression level of gibberellin and cytokinin can promote cell division and elongation, directly contributing to increased plant height and overall biomass (Chen et al., 2022; An et al., 2023). Simultaneously, the improved nitrogen assimilation process provides the necessary components for proteins and nucleic acids, while enhanced photosynthesis ensures the adequate supply of energy and carbon skeletons required for these synthesis processes (Luo et al., 2023). Within this network, 3x(p) may amplify downstream metabolic efficiency through dosage-sensitive transcription factors, potentially optimizing resource allocation and growth (Figueroa et al., 2021; Swanson-Wagner et al., 2009). This is consistent with findings in *Arabidopsis* hybrids, where heterosis is associated with improved photosynthetic capacity, including increased chlorophyll content and CO_2_ assimilation rates (Fujimoto et al., 2012; Liu et al., 2020). Our results indicate that understanding and potentially manipulating the POE-driven transcriptional reprogramming could be a powerful strategy to optimize agronomic traits, especially in perennial fruit crops like loquat where rapid and vigorous growth is highly desirable.

### 4.2 A novel epigenetic paradigm of parental allele regulation driven by POE

Besides the transcriptional changes, our ASE analysis uncovers two distinct regulatory paradigms: maternal dominance in the number of biased genes, while paternal enhancement of expression level (**Figures 1E–G**). This asymmetry implies that maternal genomes dictate allelic preference, whereas paternal genomes boost transcript levels. We propose this amplification is driven, at least in part, by dosage-dependent activation of enhancers, a mechanism consistent with observations in maize where paternal eQTLs also govern expression levels (Swanson-Wagner et al., 2009; Waters et al., 2013). Additionally, the discovery of 5 candidate parentally expressed genes (PEGs) represents an extreme form of paternal enhancement and provides strong evidence for genomic imprinting as a key contributing mechanism to POE in loquat (Waters et al., 2013; Fort et al., 2017). It is also consistent with widely observed imprinting regulatory mechanisms in angiosperms, where POE-specific DNA methylation governs allelic expression and affect dosage-sensitive pathway activity in triploids (Rodrigues et al., 2013; Batista and Köhler, 2020).

Notably, our ASM analysis uncovered a non-classical epigenetic regulatory mechanism. While promoter regions exhibited the expected negative correlation between allelic expression and methylation, paternal alleles displayed gene body hypermethylation (mCG context) that paradoxically correlates with increased expression (**Figures 4A, C**). This finding aligns with epigenetic regulatory patterns in plants like *Arabidopsis* and rice, where promoter hypomethylation acts as a transcriptional ‘switch’ for gene activation, and hypermethylation leads to silencing (Zhang et al., 2018; Zhu et al., 2018). However, this discovery also challenges the traditional view of gene body methylation (GBM) as primarily related to transcriptional stability or repression. Instead, it suggests a novel role in the expression of paternal alleles within the context of POE. This atypical hypermethylation coupling, mainly in the mCG context, was validated at single-locus levels for key functional genes (*EjTOC1* and *EjLHY*). Unlike the well-documented activation of maternal alleles in the endosperm often involving promoter demethylation by enzymes like DEMETER (DME) (Gehring, 2013; Rodrigues et al., 2013), our results point to a distinct mechanism. We hypothesize that this atypical hypermethylation-expression coupling is potentially mediated by RNA-directed DNA methylation (RdDM) pathways. The RdDM-mediated methylation could specifically target paternal alleles in gene body regions, leading to a more improved transcriptional elongation or a permissive chromatin state, and subsequently stabilizing and increasing the expression level (Wassenegger, 2000; Gehring, 2013). This is supported by observations in rice endosperm where RdDM and imprinted small RNAs contribute to paternally biased gene expression and the intricate regulation of parental effects (Rodrigues et al., 2013). Therefore, such novel epigenetic strategy of enhancing paternal transcription via gene body hypermethylation may represent a significant advancement in our understanding of POE and triploid heterosis, offering a promising new target for future polyploid breeding and genetic improvement of perennial fruit crops.

### 4.3 Circadian clock disruption contributes to enhanced seedling vigor in paternal-excess triploid loquat

A pivotal discovery from our study is the POE-dependent reprogramming of the circadian clock, positioning it as a central mediator of enhanced seedling vigor in 3x(p) loquat. As a core regulator of plant physiology, the circadian clock coordinates essential processes such as photosynthesis, starch accumulation, and hormone signaling, which are directly linked to the observed heterotic phenotypes including plant height and soluble starch content in our previous study (Izumi, 2019; Xu et al., 2022; Zhang et al., submitted for publication). Diurnal expression profiling of core clock genes (*EjCCA1*, *EjLHY*, *EjGI*, *EjTOC1*) reveals marked alterations in triploid loquat, with paternal excess driving elevated expression level, such as for *EjCCA1* at ZT18 in R2×R4, and dampened rhythms (i.e., flattened profiles of *EjTOC1*), contrasting with aberrant oscillations in 3x(m) (**Figure 5**). This disruption aligns with enriched “plant circadian rhythm” pathways in DEGs between reciprocal L2 and L4 crosses (**Figure 1D**), indicating that dosage imbalance perturbs the core feedback loop, shifting phase and amplitude to optimize growth (Hochholdinger and Yu, 2025).

In annual crops, circadian reprogramming enhances heterosis by synchronizing metabolic processes with environmental cues, a mechanism increasingly elucidated across diverse systems. For example, *Arabidopsis* allotetraploid hybrids primarily exhibit amplitude changes in clock genes (Ni et al., 2009). However, more alterations, like phase shifts, also contribute to heterosis. A phase shift model, driven by core clock transcription factors binding to carbon fixation genes, has been demonstrated in diploid maize hybrids (Ko et al., 2016). Similarly, in polyploid hybrid *Brassica napus*, sub genome dosage imbalance alters both the phase and amplitude of clock gene rhythms, disrupting feedback loops and synergizing with epigenetic modifications to optimize metabolic rhythms and fitness (Xue et al., 2023). In this study, significant shifts in both phase and amplitude have been observed in triploid loquat. Specifically, the phase shifts may reflect adaptations to subtropical environments, which allow for prolonged carbon fixation under extended photoperiods (Greenham and McClung, 2015). Paternal excess further amplifies this effect, possibly via epigenetic enhancement of clock gene expression, as shown by higher gene body mCG methylation on paternal alleles (**Figure 4C**). For instance, elevated *EjTOC1* repression in 3x(p) hybrids could extend the growth window by delaying evening repression of growth-promoting genes, paralleling *Arabidopsis* triploids where paternal dosage boosts biomass via clock-mediated starch accumulation (Ni et al., 2009; Izumi, 2019). Extending beyond annuals, this POE-driven plasticity in perennials highlights an evolutionary strategy: circadian reprogramming mitigates genomic imbalances in polyploids, promoting heterosis as an adaptive trait in variable environments (Dodd et al., 2005). This mechanism may exploit dosage effects for resilience, particularly in woody species facing climate change. Future research will track these hybrid groups to maturity to determine whether the POE-driven molecular patterns observed in seedlings are predictive of adult-stage agronomic traits, such as fruit yield, quality, and color. This long-term analysis will be critical for bridging the gap between early developmental heterosis and final performance, providing valuable markers for accelerated breeding programs.

## 5 Conclusion

This study demonstrates POE as the predominant driver of transcriptional reprogramming in triploid loquat, far exceeding the effect of ploidy or hybridity alone. At the molecular level, POE orchestrates dosage-sensitive regulatory networks, manifesting as maternal bias in ASE quantity but paternal enhancement in expression levels, including 5 paternally expressed imprinted genes. Furthermore, our ASM analysis reveals a non-classical epigenetic mechanism where paternal gene body hypermethylation (mCG) paradoxically promotes transcription, especially in circadian clock genes. The resulting reprogramming of their diurnal rhythms, validated by qRT-PCR, likely optimize metabolic efficiency and contribute to enhanced vigor in paternal-excess triploids [3x(p)].

These findings have advanced our understanding of the genetic and epigenetic basis of polyploid heterosis in perennial fruit crops, underscoring the molecular mechanisms by which POE mediates gene dosage effects and transcriptional regulation. This work opens new avenues to enhance crop performance by exploring tissue-specific imprinting, genotype-environment interactions, and manipulations of POE networks, ultimately boosting the productivity and climate resilience of loquat and other woody perennials.

## Data Availability

The datasets presented in this study can be found in online repositories. The names of the repository/repositories and accession number(s) can be found in the article/**Supplementary Material**.

## References

[B1] Ahmed D. Evrard J. C. Ollitrault P. Froelicher Y. (2020). The effect of cross direction and ploidy level on phenotypic variation of reciprocal diploid and triploid mandarin hybrids. Tree Genet. Genomes 16, 25. doi: 10.1007/s11295-020-1417-7

[B2] Aleza P. Juárez J. Cuenca J. Ollitrault P. Navarro L. (2012). Extensive citrus triploid hybrid production by 2x × 4x sexual hybridizations and parent-effect on the length of the juvenile phase. Plant Cell Rep. 31, 1723–1735. doi: 10.1007/s00299-012-1286-0, PMID: 22614256

[B3] An Y. Q. Ma D. J. Xi Z. (2023). Multi-omics analysis reveals synergistic enhancement of nitrogen assimilation efficiency via coordinated regulation of nitrogen and carbon metabolism by co-application of brassinolide and pyraclostrobin in *Arabidopsis thaliana* . Int. J. Mol. Sci. 24, 16435. doi: 10.3390/ijms242216435, PMID: 38003624 PMC10671621

[B4] Batista R. A. Köhler C. (2020). Genomic imprinting in plants—revisiting existing models. Genes Dev. 34, 24–36. doi: 10.1101/gad.332924.119, PMID: 31896690 PMC6938664

[B5] Castillo-Bravo R. Fort A. Cashell R. Brychkova G. McKeown P. C. Spillane C. (2022). Parent-of-origin effects on seed size modify heterosis responses in *Arabidopsis thaliana* . Front. Plant Sci. 13. doi: 10.3389/fpls.2022.835219, PMID: 35330872 PMC8940307

[B6] Chen M. Guo L. Ramakrishnan M. Fei Z. Vinod K. K. Ding Y.. (2022). Rapid growth of Moso bamboo (*Phyllostachys edulis)*: cellular roadmaps, transcriptome dynamics, and environmental factors. Plant Cell 34, 3577–3610. doi: 10.1093/plcell/koac193, PMID: 35766883 PMC9516176

[B7] Dodd A. N. Salathia N. Hall A. Kévei E. Tóth R. Nagy F.. (2005). Plant circadian clocks increase photosynthesis, growth, survival, and competitive advantage. Science 309, 630–633. doi: 10.1126/science.1115581, PMID: 16040710

[B8] Donoghue M. T. A. Fort A. Clifton R. Zhang X. McKeown P. C. Voigt-Zielinksi M. L.. (2014). CmCGG methylation-independent parent-of-origin effects on genome-wide transcript levels in isogenic reciprocal F1 triploid plants. DNA Res. 21, 141–151. doi: 10.1093/dnares/dst046, PMID: 24212467 PMC3989486

[B9] Feng T. Wang L. Li L. Liu Y. Chong K. Theißen G.. (2022). OsMADS14 and NF-YB1 cooperate in the direct activation of OsAGPL2 and waxy during starch synthesis in rice endosperm. New Phytol. 234, 77–92. doi: 10.1111/nph.17990, PMID: 35067957

[B10] Figueroa C. M. Lunn J. E. Iglesias A. A. (2021). Nucleotide-sugar metabolism in plants: the legacy of Luis F. Leloir. J. Exp. Bot. 72, 4053–4067. doi: 10.1093/jxb/erab109, PMID: 33948638

[B11] Filichkin S. A. Breton G. Priest H. D. Dharmawardhana P. Jaiswal P. Fox S. E.. (2011). Global profiling of rice and poplar transcriptomes highlights key conserved circadian-controlled pathways and cis-regulatory modules. PloS One 6, e16907. doi: 10.1371/journal.pone.0016907, PMID: 21694767 PMC3111414

[B12] Fort A. Ryder P. McKeown P. C. Wijnen C. Aarts M. G. Sulpice R.. (2016). Disaggregating polyploidy, parental genome dosage and hybridity contributions to heterosis in *Arabidopsis thaliana* . New Phytol. 209, 590–599. doi: 10.1111/nph.13650, PMID: 26395035

[B13] Fort A. Tuteja R. Braud M. McKeown P. C. Spillane C. (2017). Parental-genome dosage effects on the transcriptome of F1 hybrid triploid embryos of *Arabidopsis thaliana* . Plant J. 92, 1044–1058. doi: 10.1111/tpj.13740, PMID: 29024088

[B14] Friedland N. Negi S. Vinogradova-Shah T. Wu G. Ma L. Flynn S.. (2019). Fine-tuning the photosynthetic light harvesting apparatus for improved photosynthetic efficiency and biomass yield. Sci. Rep. 9, 13028. doi: 10.1038/s41598-019-49545-8, PMID: 31506512 PMC6736957

[B15] Fujimoto R. Taylor J. M. Shirasawa S. Peacock W. J. Dennis E. S. (2012). Heterosis of *Arabidopsis* hybrids between C24 and Col is associated with increased photosynthesis capacity. Proc. Natl. Acad. Sci. U. S. A. 109, 7109–7114. doi: 10.1073/pnas.1204464109, PMID: 22493265 PMC3344962

[B16] Gehring M. (2013). Genomic imprinting: insights from plants. Annu. Rev. Genet. 47, 187–208. doi: 10.1146/annurev-genet-110711-155527, PMID: 24016190

[B17] Greenham K. McClung C. R. (2015). Integrating circadian dynamics with physiological processes in plants. Nat. Rev. Genet. 16, 598–610. doi: 10.1038/nrg3976, PMID: 26370901

[B18] Hallahan B. F. Fernandez-Tendero E. Fort A. Ryder P. Dupouy G. Deletre M.. (2018). Hybridity has a greater effect than paternal genome dosage on heterosis in sugar beet (*Beta vulgaris*). BMC Plant Biol. 18, 120. doi: 10.1186/s12870-018-1338-x, PMID: 29907096 PMC6003118

[B19] Harmer S. L. Hogenesch J. B. Straume M. Chang H.-S. Han B. Zhu T.. (2000). Orchestrated transcription of key pathways in *Arabidopsis* by the circadian clock. Science 290, 2110–2113. doi: 10.1126/science.290.5499.2110, PMID: 11118138

[B20] Hochholdinger F. Yu P. (2025). Molecular concepts to explain heterosis in crops. Trends Plant Sci. 30, 95–104. doi: 10.1016/j.tplants.2024.07.018, PMID: 39191625

[B21] Hsu P. Y. Harmer S. L. (2014). Wheels within wheels: the plant circadian system. Trends Plant Sci. 19, 240–249. doi: 10.1016/j.tplants.2013.11.007, PMID: 24373845 PMC3976767

[B22] Izumi M. (2019). Roles of the clock in controlling starch metabolism. Plant Physiol. 179, 1441–1443. doi: 10.1104/pp.19.00166, PMID: 30940739 PMC6446776

[B23] Jiang W. Shi J. Zhao J. Wang Q. Cong D. Chen F.. (2021). ZFP57 dictates allelic expression switch of target imprinted genes. Proc. Natl. Acad. Sci. U. S. A. 118, e2005377118. doi: 10.1073/pnas.2005377118, PMID: 33500348 PMC7865185

[B24] Ko D. K. Rohozinski D. Song Q. Taylor S. H. Juenger T. E. Harmon F. G.. (2016). Temporal shift of circadian-mediated gene expression and carbon fixation contributes to biomass heterosis in maize hybrids. PloS Genet. 12, e1006197. doi: 10.1371/journal.pgen.1006197, PMID: 27467757 PMC4965137

[B25] Lauss K. Wardenaar R. Oka R. Van Hulten M. H. A. Guryev V. Keurentjes J. J. B.. (2018). Parental DNA methylation states are associated with heterosis in epigenetic hybrids. Plant Physiol. 176, 1627–1645. doi: 10.1104/pp.17.01054, PMID: 29196538 PMC5813580

[B26] Li C. Wen H. Wu Y. Li Y. Feng X. Li X.. (2025). OsRF2b interacting with OsbZIP61 modulates nitrogen use efficiency and grain yield via heterodimers in rice. Plant Biotechnol. J. 23, 3300–3312. doi: 10.1111/pbi.70136, PMID: 40415532 PMC12310816

[B27] Li Z. Zhao Y. Luo K. (2024). Molecular mechanisms of heterosis and its applications in tree breeding: progress and perspectives. J. Mol. Sci. 25, 12344. doi: 10.3390/ijms252212344, PMID: 39596408 PMC11594601

[B28] Lin S. Q. Sharpe R. H. Janick J. (1998). Loquat: botany and horticulture. Hortic. Rev. 23, 233–276. doi: 10.1002/9780470650752.ch5

[B29] Liu C. Huang R. Wang L. Liang G. (2021). Functional identification of EjGIF1 in *Arabidopsis* and preliminary analysis of its regulatory mechanisms in the formation of triploid loquat leaf heterosis. Front. Plant Sci. 11. doi: 10.3389/fpls.2020.612055, PMID: 33510754 PMC7835675

[B30] Liu C. Liu T. Ohlson E. W. Wang L. Wu D. Guo Q.. (2019). Loquat (*Eriobotrya japonica* (Thunb.) circadian clock gene cloning and heterosis studies of artificial triploid loquat. Sci. Hortic. 246, 328–337. doi: 10.1016/j.scienta.2018.10.068

[B31] Liu P. C. Peacock W. J. Wang L. Furbank R. Larkum A. Dennis E. S. (2020). Leaf growth in early development is key to biomass heterosis in. Arabidopsis. J. Exp. Bot. 71, 2439–2450. doi: 10.1093/jxb/eraa006, PMID: 31960925 PMC7178430

[B32] Lu A. Zeng S. Pi K. Long B. Mo Z. Liu R. (2024). Transcriptome analysis reveals the key role of overdominant expression of photosynthetic and respiration-related genes in the formation of tobacco(*Nicotiana tabacum* L.) biomass heterosis. BMC Genomics 25, 598. doi: 10.1186/s12864-024-10507-8, PMID: 38877410 PMC11177473

[B33] Luo J. Hang J. Wu B. Wei X. Zhao Q. Fang Z. (2023). Co-overexpression of genes for nitrogen transport, assimilation, and utilization boosts rice grain yield and nitrogen use efficiency. Crop J. 11, 785–799. doi: 10.1016/j.cj.2023.01.005

[B34] Miller M. Zhang C. Chen Z. J. (2012). Ploidy and hybridity effects on growth vigor and gene expression in *Arabidopsis thaliana* hybrids and their parents. G3:Genes Genomes Genet. 2, 505–513. doi: 10.1534/g3.112.002162, PMID: 22540042 PMC3337479

[B35] Ng D. W. K. Miller M. Yu H. H. Huang T. Y. Kim E. D. Lu J.. (2014). A role for CHH methylation in the parent-of-origin effect on altered circadian rhythms and biomass heterosis in *Arabidopsis* intraspecific hybrids. Plant Cell 26, 2430–2440. doi: 10.1105/tpc.113.115980, PMID: 24894042 PMC4114943

[B36] Ni Z. Kim E.-D. Ha M. Lackey E. Liu J. Zhang Y.. (2009). Altered circadian rhythms regulate growth vigour in hybrids and allopolyploids. Nature 457, 327–331. doi: 10.1038/nature07523, PMID: 19029881 PMC2679702

[B37] Reik W. Walter J. (2001). Genomic imprinting: parental influence on the genome. Nat. Rev. Genet. 2, 21–32. doi: 10.1038/35047554, PMID: 11253064

[B38] Rodrigues J. A. Ruan R. Nishimura T. Sharma M. K. Sharma R. Ronald P. C.. (2013). Imprinted expression of genes and small RNA is associated with localized hypomethylation of the maternal genome in rice endosperm. Proc. Natl. Acad. Sci. U. S. A. 110, 7934–7939. doi: 10.1073/pnas.1306164110, PMID: 23613580 PMC3651473

[B39] Sedov E. N. Sedysheva G. A. Serova Z. M. Gorbacheva N. G. Melnik S. A. (2014). Breeding assessment of heteroploid crosses in the development of triploid apple varieties. Russ. J. Genet.: Appl. Res. 4, 52–59. doi: 10.1134/S2079059714010109

[B40] Singh A. Roychoudhury A. (2022). “Mechanism of crosstalk between cytokinin and gibberellin,” in Auxins, cytokinins and gibberellins signaling in plants. Ed. Aftab T. (Springer International Publishing, Cham), 77–90. doi: 10.1007/978-3-031-05427-3_4

[B41] Su W. Jing Y. Lin S. Yue Z. Yang X. Xu J.. (2021). Polyploidy underlies co-option and diversification of biosynthetic triterpene pathways in the apple tribe. Proc. Natl. Acad. Sci. U. S. A. 118, e2101767118. doi: 10.1073/pnas.2101767118, PMID: 33986115 PMC8157987

[B42] Swanson-Wagner R. A. DeCook R. Jia Y. Bancroft T. Ji T. Zhao X.. (2009). Paternal dominance of trans-eQTL influences gene expression patterns in maize hybrids. Science 326, 1118–1120. doi: 10.1126/science.1178294, PMID: 19965432

[B43] Takuno S. Gaut B. S. (2012). Body-methylated genes in *Arabidopsis thaliana* are functionally important and evolve slowly. Mol. Biol. Evol. 29, 219–227. doi: 10.1093/molbev/msr188, PMID: 21813466

[B44] Wang H. Beyene G. Zhai J. Feng S. Fahlgren N. Taylor N. J.. (2015). CG gene body DNA methylation changes and evolution of duplicated genes in cassava. Proc. Natl. Acad. Sci. U. S. A. 112, 13729–13734. doi: 10.1073/pnas.1519067112, PMID: 26483493 PMC4640745

[B45] Wassenegger M. (2000). RNA-directed DNA methylation. Plant Mol. Biol. 43, 203–220. doi: 10.1023/A:1006479327881, PMID: 10999405

[B46] Waters A. J. Bilinski P. Eichten S. R. Vaughn M. W. Ross-Ibarra J. Gehring M.. (2013). Comprehensive analysis of imprinted genes in maize reveals allelic variation for imprinting and limited conservation with other species. Proc. Natl. Acad. Sci. U. S. A. 110, 19639–19644. doi: 10.1073/pnas.1309182110, PMID: 24218619 PMC3845156

[B47] Wu X. Liu Y. Zhang Y. Gu R. (2021). Advances in research on the mechanism of heterosis in plants. Front. Plant Sci. 12. doi: 10.3389/fpls.2021.745726, PMID: 34646291 PMC8502865

[B48] Xu X. Yang H. Suo X. Liu M. Jing D. Zhang Y.. (2023). EjFAD8 enhances the low-temperature tolerance of loquat by desaturation of sulfoquinovosyl diacylglycerol (SQDG). Int. J. Mol. Sci. 24, 6946. doi: 10.3390/ijms24086946, PMID: 37108110 PMC10138649

[B49] Xu X. Yuan L. Yang X. Zhang X. Wang L. Xie Q. (2022). Circadian clock in plants: Linking timing to fitness. J. Integr. Plant Biol. 64, 792–811. doi: 10.1111/jipb.13230, PMID: 35088570

[B50] Xue Z. Gao B. Chen G. Liu J. Ouyang W. Foda M. F.. (2023). Diurnal oscillations of epigenetic modifications are associated with variation in rhythmic expression of homoeologous genes in *Brassica napus* . BMC Biol. 21, 241. doi: 10.1186/s12915-023-01735-7, PMID: 37907908 PMC10617162

[B51] Yao H. Dogra Gray A. Auger D. L. Birchler J. A. (2013). Genomic dosage effects on heterosis in triploid maize. Proc. Natl. Acad. Sci. U. S. A. 110, 2665–2669. doi: 10.1073/pnas.1221966110, PMID: 23359717 PMC3574931

[B52] Zhang H. Ali A. Hou F. Wu T. Guo D. Zeng X.. (2018). Effects of ploidy variation on promoter DNA methylation and gene expression in rice (*Oryza sativa* L.). BMC Plant Biol. 18, 314. doi: 10.1186/s12870-018-1553-5, PMID: 30497392 PMC6267922

[B53] Zhang M. Tang Y. W. Qi J. Liu X. K. Yan D. F. Zhong N. S.. (2019). Effects of parental genetic divergence on gene expression patterns in interspecific hybrids of *Camellia* . BMC Genomics 20, 828. doi: 10.1186/s12864-019-6222-z, PMID: 31703692 PMC6842218

[B54] Zhao L. Liu F. Crawford N. M. Wang Y. (2018). Molecular regulation of nitrate responses in plants. Int. J. Mol. Sci. 19, 2039. doi: 10.3390/ijms19072039, PMID: 30011829 PMC6073361

[B55] Zhu H. Xie W. Xu D. Miki D. Tang K. Huang C. F.. (2018). DNA demethylase ROS1 negatively regulates the imprinting of DOGL4 and seed dormancy in *Arabidopsis thaliana* . Proc. Natl. Acad. Sci. U. S. A. 115, E9962–E9970. doi: 10.1073/pnas.1812847115, PMID: 30266793 PMC6196528

