# Supplementary material for: Parent-of-origin effects orchestrate transcriptional reprogramming and epigenetic regulation of seedling vigor heterosis in triploid loquat
Source: Front Plant Sci. 2025 Oct 20;16:1698577. doi: 10.3389/fpls.2025.1698577 (PMC12580266; doi:10.3389/fpls.2025.1698577)
Supplement: Supplementary file 1 [file Supplementaryfile1.docx]

Supplementary Material

# Supplementary Figures


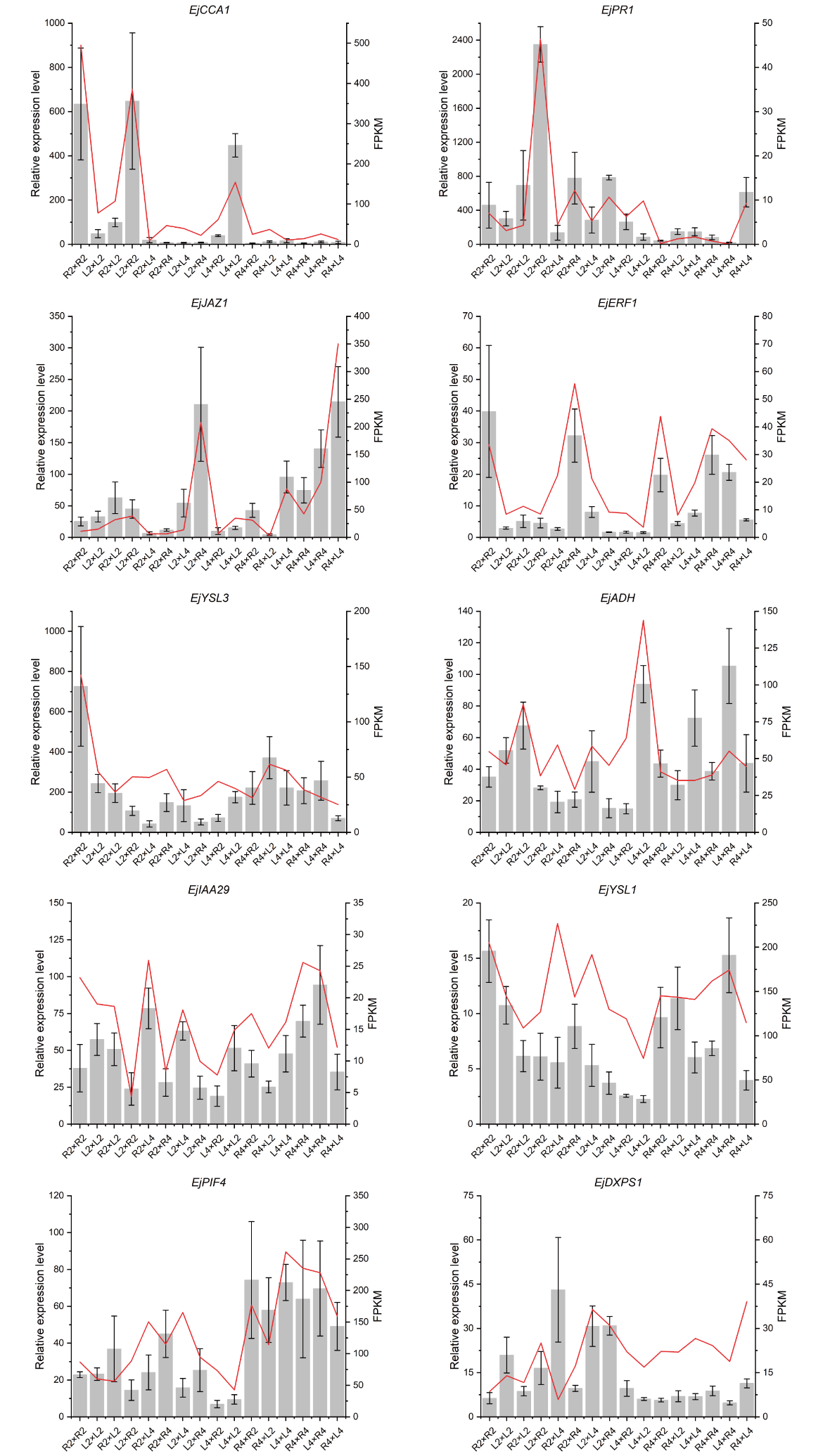


**Supplementary Figure 1.** Experimental validation of RNA-seq data by qRT-PCR. the bar chart are qRT-PCR analyses of DEGs in different crosses. The line images are changes in transcription abundance, which were detected by FPKM.
